# Efficacy and safety of cinepazide maleate injection in patients with acute ischemic stroke: a multicenter, randomized, double-blind, placebo-controlled trial

**DOI:** 10.1186/s12883-020-01844-8

**Published:** 2020-07-14

**Authors:** Jun Ni, Huisheng Chen, Guofang Chen, Yong Ji, Fei Yi, Zhuobo Zhang, Yi Yang, Jin Wu, Xueli Cai, Bei Shao, Jianfeng Wang, Yafang Liu, Deqin Geng, Xinhui Qu, Xiaohong Li, Yan Wei, Jianping Ding, Hua Lü, Yining Huang, Yonghua Huang, Bo Xiao, Tao Gong, Liying Cui, on behalf of the study collaboration group, Dong Wang, Shugen Han, Xiaoping Gao, Xiaorong Zhuang, Guojun Tan, Runxiu Zhu, Hongye Bi, Hong Yang, Youqing Deng, Jinghua Zhou, Shengzhe Zheng, Zhiyong Wang, Xiaodong Lu, Juntao Li, Lina Huang, Weimin Hu, Dawei Zang, Xiaoxi Yao, Li Li, Liandong Zhao, Luoqing Li, Shifang Wang, Kaifu Ke, Tianming Lu, Qilin Ma, Qing Zhang, Baojun Wang, Liang Zhao, Hongliang Dong, Wei Gao, Ying Liu, Yamei Tang, Junfeng Gao, Xiaofei Yu, Libin Guo, Haiyan Lin, Xiue Wei, Chenglin Tian, Tong Zhang, Yaguo Li, Guoqiang Wen, Chengfang Zhou, Qi Fang

**Affiliations:** 1 Peking Union Medical College Hospital, Chinese Academy of Medical Sciences, No.1 Shuaifuyuan Wangfujing Dongcheng District, Beijing, 100730 China; 2 General Hospital of Northern Theater Command, Shenyang, China; 3 Xuzhou Central Hospital, Xuzhou, China; 4 Tianjin Huanhu Hospital, Tianjin, China; 5 Pingxiang People’s Hospital, Pingxiang, China; 6 Fourth Affiliated Hospital of Harbin Medical University, Harbin, China; 7 First Bethune Hospital of Jilin University, Changchun, China; 8 Second Affiliated Hospital of Nanjing Medical University, Nanjing, China; 9 Lishui Municipal Central Hospital, Lishui, China; 10 First Affiliated Hospital of Wenzhou Medical University, Wenzhou, China; 11 Dalian Municipal Central Hospital, Dalian, China; 12 Huangshi Central Hospital, Huangshi, China; 13 Affiliated Hospital of Xuzhou Medical University, Xuzhou, China; 14 Jiangxi Provincial People’s Hospital affiliated to Nanchang University, Nanchang, China; 15 Jinan Central Hospital, Jinan, China; 16 Hengshui People’s Hospital (Harrison International Peace Hospital), Hengshui, China; 17 Xuan Wu Hospital, Capital Medical University, Beijing, China; 18 Shaanxi Provincial People’s Hospital, Xi’an, China; 19 Peking University First Hospital, Beijing, China; 20 Seventh Medical Center of the Chinese PLA General Hospital, Beijing, China; 21 Xiangya Hospital Central South University, Changsha, China; 22 Beijing Hospital, Beijing, China

**Keywords:** Acute cerebral infarction, Cerebrovascular disease, Cinepazide maleate, Stroke

## Abstract

**Background:**

Ischemic stroke is a leading cause of morbidity and mortality. Thrombolytic therapy improves disability and survival rates; however, to be effective, it must be given within 4.5 h of onset. Moreover, thrombolytic therapy is frequently contraindicated. Therefore, alternative therapeutic options are required. In China, cinepazide maleate injection has been shown to improve the cerebral collateral circulation and further reduce disability in stroke patients; however, very few studies investigating this therapy have been conducted to date. Therefore, this study aimed to further confirm the efficacy and safety of cinepazide maleate injection in patients with acute ischemic stroke.

**Methods:**

Patients with acute ischemic stroke were administered an intravenous infusion of 320 mg cinepazide maleate or placebo once daily for 14 days. All patients were also administered basic therapy (citicoline sodium). The primary efficacy endpoint was the proportion of patients with a modified Rankin scale (mRS) ≤2 on day 90. Secondary efficacy endpoints included Barthel Index ≥95. Safety was evaluated by recording all adverse events (AEs), monitoring laboratory parameters and vital signs, and electrocardiogram.

**Results:**

In total, 937 patients with an acute ischemic stroke were included, with a mean (standard deviation, SD) National Institutes of Health Stroke Scale score of 8.8 (2.4) and a mean (SD) stroke onset of 30.9 (11.4) hours prior. Following treatment for 90 days, the proportion of patients with an mRS score ≤ 2 was significantly higher in the cinepazide maleate group than in the control group (60.9% vs. 50.1%; *p* = 0.0004). Moreover, the proportion of patients with a Barthel Index of ≥95 on day 90 was also significantly higher in the cinepazide maleate group than in the control group (53.4% vs. 46.7%; *p* = 0.0230). There were no statistically significant differences in safety parameters between the cinepazide maleate and control groups.

**Conclusions:**

The results of this study show that cinepazide maleate injection is superior to placebo in improving neurological function and activities of daily living, reducing disability, and promoting functional recovery in patients with acute ischemic stroke. Cinepazide maleate injection was safe and well tolerated with no unexpected AEs reported.

**Trial registration:**

Chinese Clinical Trial Registry CTR20160292 and ChiCTR1900023827. Retrospectively registered June 13, 2019.

## Background

Ischemic stroke is an acute cerebrovascular event caused by decreased blood flow to the brain. The long-term effects of stroke include decreased quality of life and a high rate of morbidity and mortality [1]. In recent years, ischemic stroke has been shown to be the leading cause of death in China, which has the highest stroke incidence (247/100,000) and stroke mortality (115/100,000) rates in the world [2]. The most common stroke subtype in China is ischemic stroke, which accounts for 69.6% of all stroke events [2].

Ischemic stroke is categorized into five subtypes based on etiology, as defined in the Trial of Org 10,172 in Acute Stroke Treatment (TOAST) classification: large-artery atherosclerosis; cardioembolism; small-vessel occlusion; stroke of other determined etiology; and stroke of undetermined etiology [3]. Stroke etiology has a major influence on prognosis; therefore, the correct treatment strategy requires a rapid assessment followed by early diagnosis and intervention with a thrombolytic agent to minimize functional disability caused by nerve damage [4]. Thrombolytic therapy after acute ischemic stroke reduces morbidity and mortality rates and improves the quality of life of patients, but only if the patient is correctly diagnosed within 4.5 h of onset [5]. However, many patients are not diagnosed within this time frame, and even among those who are, many are contraindicated for thrombolytic therapy [6]. Therefore, other effective therapeutic options are required.

Cinepazide maleate is a piperazine derivative that acts as a weak calcium channel blocker and has been shown to reduce disability following acute ischemic stroke [7]. Cinepazide maleate potentiates the effects of increased endogenous adenosine in atrial tissues, retards the degradation of adenosine, inhibits platelet aggregation, reduces blood viscosity, and improves blood rheology [7–11]. A tablet formulation of cinepazide maleate was originally approved in 1974 but was withdrawn in the 1990s owing to an apparent increased risk of agranulocytosis. A cinepazide maleate injection was later (in 2002) approved in China, where it is widely used for the treatment of acute ischemic stroke, the sequelae of brain trauma, unstable angina, and other cerebrovascular diseases [12–15]. Given the lack of good quality data on the efficacy of cinepazide maleate injection for treating ischemic stroke, the high incidence of ischemic stroke (and high mortality owing to ischemic stroke) in China, and the continued widespread use of cinepazide maleate injection in China, the present study was designed to evaluate and further confirm the efficacy and safety of cinepazide maleate injection for the treatment of patients after acute ischemic stroke.

## Methods

### Study design and treatment

**Fig. 1:**
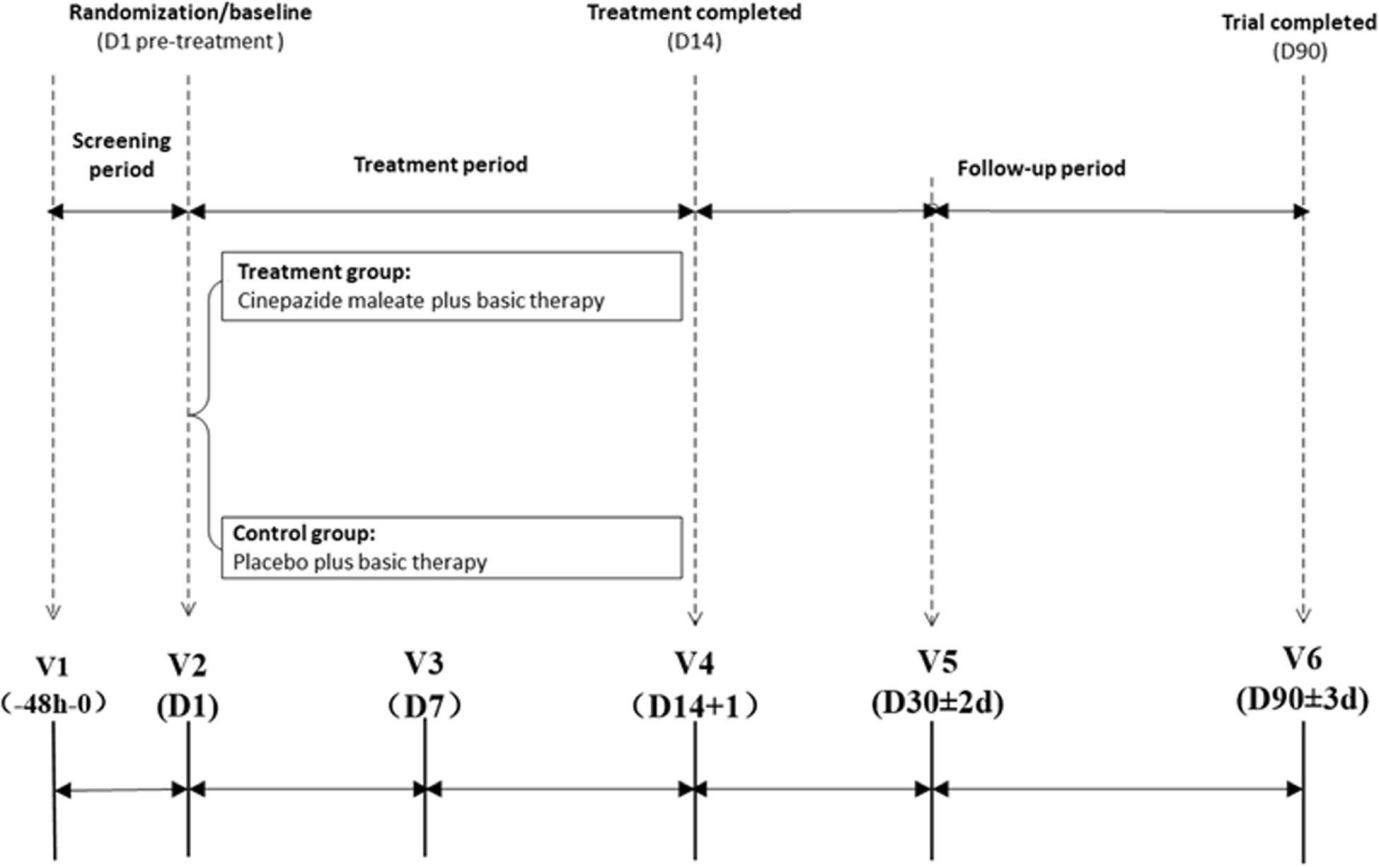
Study design. Patients were assessed during the screening period and then on day 1 (baseline), day 7, and day 14 of the treatment period. After 14 days, patients entered a follow-up period for an additional 75 days and the study was completed on day 90 (±3 days). During the follow-up period, patients were assessed on days 30 and 90. D, day; V, visit

This multicenter, randomized, double-blind, parallel-group, placebo-controlled, post-market clinical trial aimed to evaluate the safety and efficacy of cinepazide maleate injection in Chinese patients with acute ischemic stroke (Fig. 1). This study was conducted across 72 medical centers (of which 65 enrolled patients and 60 treated patients with cinepazide maleate; five centers had only patients randomly assigned no drug treatment) in China from August 2016 to February 2019 and was registered in the Chinese Clinical Trial Registry (registration numbers: CTR20160292 and ChiCTR1900023827). The ethics committees of all 72 research institutes reviewed and approved the study protocol. The study was conducted in accordance with the ethical guidelines for human medical research as stated in the Declaration of Helsinki (2013) and the ethical principles of the Chinese Good Clinical Practice for Drug Administration. All patients provided voluntary written informed consent prior to commencing any study procedures.

Patients received a continuous intravenous infusion of 320 mg of cinepazide maleate (in 500 mL of saline) or placebo (saline alone) at a rate of 100 mL/hour once daily for 14 days. Patients also received basic therapy, consisting of an intravenous infusion of 250 mL of citicoline sodium (0.5 g dissolved in 5% glucose or saline) once daily for 10 days. Furthermore, in accordance with the Chinese Guidelines on the Diagnosis and Treatment of Acute Ischemic Stroke in 2014 [16], all patients were allowed to receive antihypertensive, antihyperlipidemic, and anticoagulant agents together with agents to lower blood sugar and mannitol to reduce intracranial pressure.

Patients were assessed during the screening period and then on day 1 (baseline), day 7, and day 14 of the treatment period. After 14 days, patients entered a follow-up period for an additional 75 days and the study was completed on day 90 (±3 days). During the follow-up period, patients were assessed on day 30 and day 90.

### Randomization and blinding

This study used an interactive web response system for randomization and both patients and investigators were blinded throughout the study.

### Patients

Initially, this study included patients with National Institutes of Health Stroke Scale (NIHSS) scores of 5–25 at first diagnosis. However, the entry criteria were revised to patients with NIHSS scores of 7–25 for the following reasons. First, in February 2018, the Chinese Food and Drug Administration issued guidelines for clinical trials of therapeutic drugs for acute ischemic stroke in which they pointed out that baseline severity may influence the outcome of clinical trials; therefore, the inclusion criteria were revised to limit the population to patients with moderate neurological impairment at baseline. Thus, based on our definition of mild neurological impairment, patients whose symptoms were classified as NIHSS 1–6 were excluded because of their high self-recovery capacity. In addition, the TOAST trial [17] evaluated the outcomes of 1281 acute ischemic stroke patients, and the results suggested that those with NIHSS scores ≥16 at baseline tended to have a poor prognosis and those with NIHSS scores ≤6 at baseline tended to have better outcomes. Furthermore, during an interim re-estimation of the required sample size for this study, it was shown that, of the 533 patients who completed the last visit, up to 87.6% (311/355 patients) of those who had an NIHSS score between 5 and 6 at baseline had a modified Rankin scale (mRS) score of ≤2 at day 90. This finding suggested that patients with baseline NIHSS scores between 5 and 6 have a strong self-recovery capacity.

Finally, the International Conference on Harmonization E9 guideline and Food and Drug Administration Guidance for Industry recommend changing the design of long-term clinical studies if considered appropriate based on new information or increased medical knowledge [18, 19]. Although patients with an NIHSS score of 5 or 6 were initially included in the patient population, these patients were then excluded from the current FAS analysis.

In this study, the patient population included those who had experienced an ischemic stroke within the previous 48 h prior to study entry. Inclusion criteria were age 18–80 years, diagnosed with either a first-time acute internal carotid artery (anterior circulation) stroke or a recurrent stroke with a good prognosis (mRS score of 0–1) before time of relapse, and an NIHSS score of 7–25.

Exclusion criteria were: among patients with recurrent stroke, an mRS score of ≥2 before onset of the most recent stroke; a cranial computed tomography scan indicating an intracerebral hemorrhage (e.g., hemorrhagic stroke, epidural hematoma, intracerebral hematoma, intraventricular hemorrhage, and subarachnoid hemorrhage); cerebral infarction accompanied by disorders of consciousness, transient ischemic attack, cerebral arteritis, brain tumor(s), traumatic brain injury, intracranial infection, or brain parasites; poorly controlled hypertension (systolic blood pressure ≥ 200 mmHg or diastolic blood pressure ≥ 110 mmHg); high risk of cardiac embolism, acute myocardial infarction, or heart failure; bleeding tendency or a history of severe bleeding within the past 3 months; epilepsy; malignant tumor or a severe and progressive disease; and presence of a psychiatric disorder that may lead to poor compliance.

The following treatments were prohibited during the study: any interventional therapy, including thrombolytic therapy; fibrinogen-depleting therapy; platelet inhibitors except for aspirin and clopidogrel; cerebral vasodilators (e.g., cinnarizine, flunarizine, nicardipine, nimodipine); neuroprotective agents (e.g., edaravone, piracetam, monosialoganglioside sodium); drugs that improve cerebral circulation; or any traditional Chinese medicines with the indication of promoting blood circulation, removing blood stasis, or with the indication of treating cerebral infarction.

### Outcomes

The primary efficacy endpoint was the percentage of patients with an mRS score of ≤2 on day 90. Secondary efficacy endpoints included the percentage of patients with a Barthel Index score of ≥95 on days 14, 30, and 90; we report data for day 90 only here.

Safety was evaluated by monitoring adverse events (AEs), monitoring laboratory parameters (routine blood test, urine test, biochemical examinations, coagulation profile, and lipid profile) and vital signs, and electrocardiogram (parameters analyzed were heart rate, PR interval, QTc, and QRS intervals).

### Statistical analysis

The sample size calculation was based on the estimated number of patients that would achieve a 90-day mRS score of ≤2 points. Based on a previous study [20] and observations from clinical practice, we estimated the number of patients that would achieve a 90-day mRS score of ≤2 points in the experimental and placebo groups to be 45 and 35%, respectively. Thus, it was calculated that a sample size of 596 patients in each group would ensure an 80% power to detect differences between groups at a two-sided significance level of 0.05. To allow for the exclusion of patients who had joined the trial with NIHSS scores of 5 and 6 at baseline (revision to inclusion criteria after the study commenced), and for the loss of patients during the study owing to withdrawal, the sample size required was estimated to be 1300 patients.

Patients who had an NIHSS score ≥ 7 at the time of screening and were subsequently randomized for treatment were included in the full analysis set. To be included in the safety analysis set, patients were required to have received treatment after randomization.

Descriptive statistics were used to summarize patient demographics and clinical characteristics at baseline. A logistic regression analysis was used to determine the difference in efficacy endpoints between the groups, and a *p*-value of < 0.05 was considered to show a statistically significant difference. Odds ratios (OR) were obtained using a logistic regression model with the treatment regimen, baseline NIHSS score, and onset time as covariates. An OR of < 1 indicated a reduced risk. Changes from one mRS point category to another were assessed using independent sample rank sum tests. A p-value of < 0.05 was considered to show a statistically significant difference.

The statistical software used was SAS version 9.4 (SAS Institute Inc. Cary, NC, USA).

## Results

### Patients

**Fig. 2:**
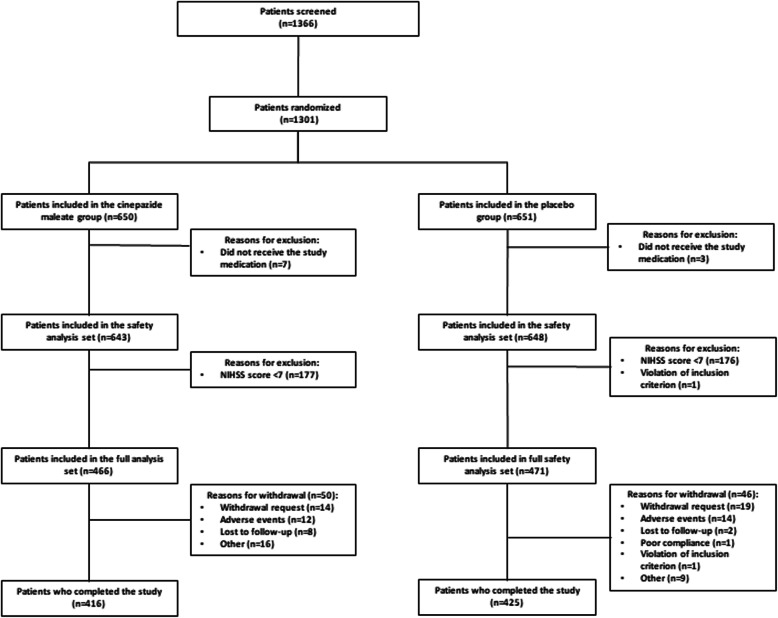
Study population and flow through the study

In this study, a total of 1366 patients were screened, of which 1301 patients were randomized to either the cinepazide maleate group (*n* = 650) or the control group (*n* = 651) (Fig. 2). Of these, 937 patients with an NIHSS score of 7–25 were included in the full analysis set (cinepazide maleate group, *n* = 466; control group, *n* = 471), and 1291 patients were included in the safety analysis set (cinepazide maleate group, *n* = 643; control group, *n* = 648).

Baseline characteristics and key clinical characteristics of patients included in this study are shown in Table 1; generally, these were well balanced between groups. In brief, the mean (standard deviation, SD) onset of cerebral infarction was 30.9 (11.4) hours prior to receiving the first study drug. Patients were recorded as having an mRS score at baseline of 0 (0.0%), 1 (0.1%), 2 (1.4%), 3 (31.7%), 4 (62.1%), or 5 (4.7%). In this study, 66.3% of patients were male and the mean (SD) body mass index was 24.3 (3.3) kg/m^2^. The mean (SD) age was 61.2 (10.0) years. There was a statistically significant difference in age between the cinepazide maleate group (60.3 [10.31]) and the placebo group (62.1 [9.65]); thus, we also performed analyses after making an adjustment to endpoint values based on this difference. Baseline NIHSS scores were 7–9 points in 694 subjects, 10–14 points in 212 subjects, 15–10 points in 28 subjects, and 20–25 points in three subjects. Furthermore, 94.6% of patients were classified as Han Chinese.

**Table 1:** Baseline characteristics of patients

	Cinepazide maleate (*n* = 466)	Placebo (*n* = 471)	Total (*n* = 937)
Age, years, mean (SD)	60.3 (10.31)	62.1 (9.65)	61.2 (10.02)
Sex, n (%)			
Male	312 (67.0)	309 (65.6)	621 (66.3)
Female	154 (33.1)	162 (34.4)	316 (33.7)
Ethnicity, n (%)			
Han	443 (95.1)	443 (94.1)	886 (94.6)
Others	23 (4.9)	28 (5.9)	51 (5.4)
BMI, kg/m^2^, mean (SD)	24.4 (3.38)	24.2 (3.30)	24.3 (3.34)
Onset of therapy, n (%)			
< 12 h	24 (5.2)	30 (6.4)	54 (5.8)
12–24 h	122 (26.2)	119 (25.3)	241 (25.7)
25–48 h	316 (67.8)	319 (67.7)	635 (67.8)
> 48 h	4 (0.9)	3 (0.6)	7 (0.8)
NIHSS score, median (Q1, Q3)	8 (7, 10)	8 (7, 10)	8 (7, 10)
mRS level, n (%)			
Level 0	0 (0.0)	0 (0.0)	0 (0.0)
Level 1	0 (0.0)	1 (0.2)	1 (0.1)
Level 2	7 (1.5)	6 (1.3)	13 (1.4)
Level 3	136 (29.2)	161 (34.2)	297 (31.7)
Level 4	303 (65.0)	279 (59.2)	582 (62.1)
Level 5	20 (4.3)	24 (5.1)	44 (4.7)
Prior history of stroke, n (%)	141 (30.3)	136 (28.9)	277 (29.6)
Comorbid disorders, n (%)			
Hypertension	348 (74.7)	343 (72.8)	691 (73.8)
Hyperlipidemia	161 (34.6)	156 (33.1)	317 (33.8)
Diabetes	145 (31.1)	160 (34.0)	305 (32.6)
Carotid atherosclerosis	105 (22.5)	128 (27.2)	233 (24.9)
Cerebral infarction	110 (23.6)	107 (22.7)	217 (23.2)
Cerebral artery stenosis	90 (19.3)	79 (16.8)	169 (18.0)
Cerebral arteriosclerosis	83 (17.8)	82 (17.4)	165 (17.6)
Carotid thrombosis	82 (17.6)	69 (14.7)	151 (16.1)
Arteriosclerosis	65 (14.0)	80 (17.0)	145 (15.5)
Hyperhomocysteinemia	65 (14.0)	71 (15.1)	136 (14.5)
Hepatic steatosis	60 (12.9)	63 (13.4)	123 (13.1)
Dyslipidemia	46 (9.9)	53 (11.3)	99 (10.6)
Coronary artery disease	51 (10.9)	43 (9.1)	94 (10.0)

Onset of therapy, time from onset to first dose

*BMI* body mass index, *mRS* modified Rankin scale, *NIHSS* National Institutes of Health Stroke Scale, *SD* standard deviation

### Primary outcome

**Fig. 3:**
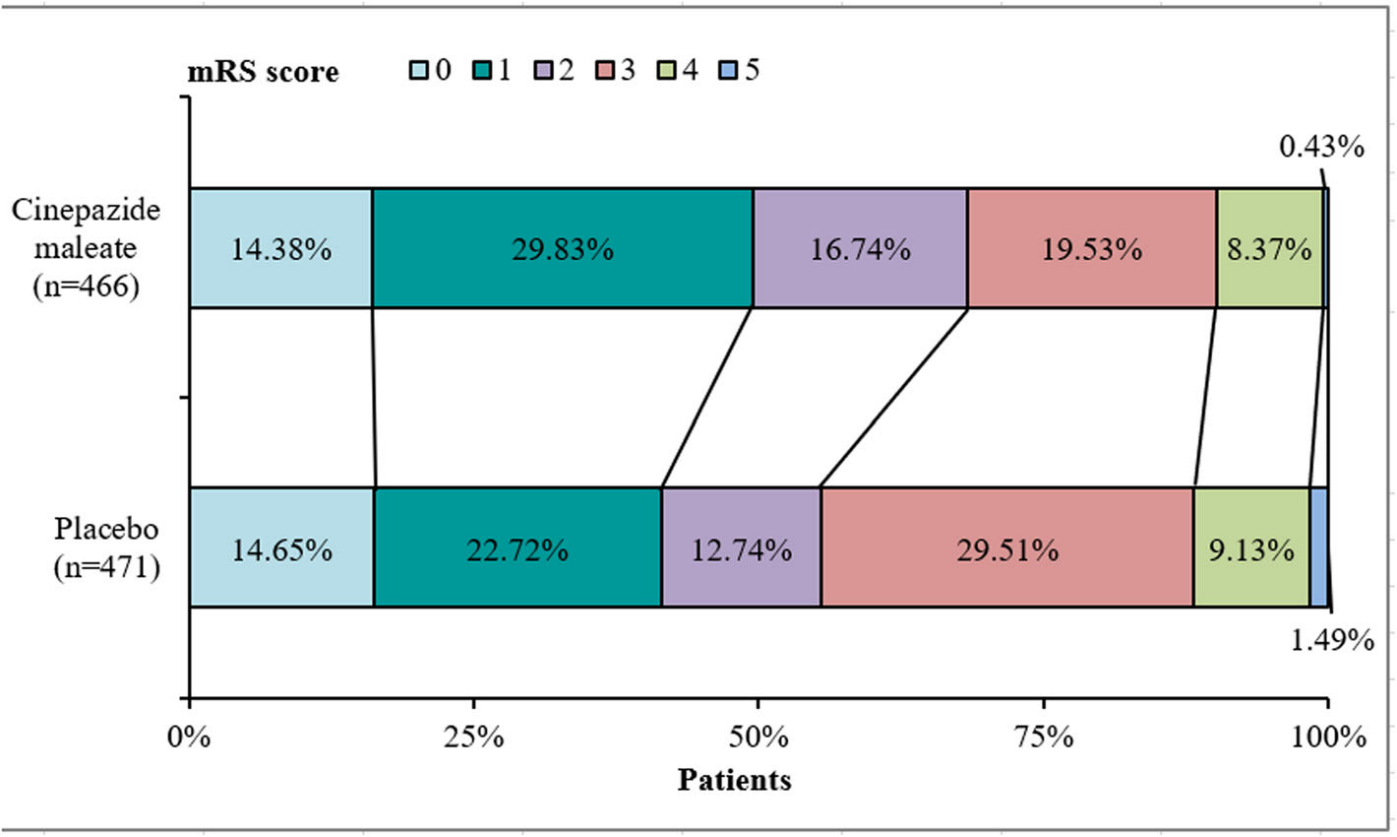
Distribution of mRS scores on day 90 among patients in the full analysis set. Proportion of patients in each mRS score category (0 to 5) by treatment group. mRS, modified Rankin Scale

There was a significant difference between the cinepazide maleate group and the control group in the proportion of patients in the full analysis set with an mRS score ≤ 2 on day 90 (60.9% vs. 50.1%, *p* = 0.0004; *p* = 0.001 when data were further adjusted for age) (Table 2). Compared with the control group, the odds ratio for a patient in the cinepazide maleate group having an mRS score > 2 on day 90 was 0.607 (95% confidence interval [CI]: 0.460, 0.801). This difference was maintained after adjusting for possible center effects as well as baseline NIHSS score and time from onset to administration of treatment in a sensitivity analysis, with an effective percent difference of 10.76% (standard error, 3.155%; 95% CI 4.57–16.94) in favor of cinepazide maleate. In addition, the overall distribution of mRS scores on day 90 showed a significant effect that favored the administration of cinepazide maleate injection over placebo (*p* = 0.009) (Table 2; Fig. 3).

**Table 2:** Distribution of mRS scores on day 90 after treatment (full analysis set)

	Cinepazide maleate (*n* = 466)	Placebo (*n* = 471)	*P*	Z
mRS score at 90 days			0.009	−2.595
Level 0	67 (14.4)	69 (14.7)	–	–
Level 1	139 (29.8)	107 (22.7)	–	–
Level 2	78 (16.7)	60 (12.7)	–	–
Level 3	91 (19.5)	139 (29.5)	–	–
Level 4	39 (8.4)	43 (9.1)	–	–
Level 5	2 (0.4)	7 (1.5)	–	–
≤ level 2	284 (60.9)	236 (50.1)	0.0004	0.607 (0.460, 0.801)^a^
Missing (did not complete the study)	50 (10.7)	46 (9.8)		

Data are presented as n (%)

*CI* confidence interval, *mRS* modified Rankin scale, *Z* Z statistic

*p* value and Z statistic are from two independent sample rank sum tests

^a^Odds ratio (95% confidence interval)

### Secondary outcome

The proportion of patients with a Barthel Index of ≥95 on day 90 was significantly higher in the cinepazide maleate group than in the control group (53.4% vs. 46.7%, *p* = 0.0230; *p* = 0.012 when data were further adjusted for age). As such, when compared with the control group, patients in the cinepazide maleate group had a lower risk of a Barthel Index < 95 on day 90 (OR = 0.719; 95% CI: 0.542, 0.956).

### Adverse events

In the cinepazide maleate and control groups, respectively, 82.0 and 84.1% of patients reported an AE (Table 3). The most common AE reported in the cinepazide maleate and control groups was constipation (26.0 and 26.5%, respectively), with no statistical difference (*p* = 0.82). The incidence of hypokalemia differed significantly between the groups, being 6.1% in the cinepazide maleate group and 10.5% in the control group (*p* = 0.0004), but this difference was not thought to be related to the investigational product. AEs occurring with an incidence ≥5% are listed in Table 3. Other than hypokalemia, none of these differed significantly between the two groups.

**Table 3:** Safety profile summary and most common (frequency ≥ 5%) adverse events (safety analysis set)

	Cinepazide maleate (*n* = 643)	Placebo (*n* = 648)	Total (*n* = 1291)	*P* value for the comparison between groups
All adverse events	527 (82.0)	545 (84.1)	1072 (83.0)	0.30
Drug-related adverse events	61 (9.5)	79 (12.2)	140 (10.8)	0.12
Serious adverse events	62 (9.6)	74 (11.4)	136 (10.5)	0.30
Drug-related serious adverse events	2 (0.3)	1 (0.2)	3 (0.2)	0.99
Adverse events leading to discontinuation	11 (1.7)	15 (2.3)	26 (2.0)	0.44
Adverse events leading to death	10 (1.6)	13 (2.0)	23 (1.8)	0.54
**Adverse events occurring with a frequency ≥ 5% (total)**	345 (53.7)	365 (56.3)	710 (55.0)	0.33
**Gastrointestinal diseases**	167 (26.0)	172 (26.5)	339 (26.3)	0.82
Constipation	167 (26.0)	172 (26.5)	339 (26.3)	0.82
**Infectious diseases**	85 (13.2)	97 (15.0)	182 (14.1)	0.37
Upper respiratory tract infection	29 (4.5)	42 (6.5)	71 (5.5)	0.12
Urinary tract infection	31 (4.8)	37 (5.7)	68 (5.3)	0.47
Pulmonary infection	30 (4.7)	36 (5.6)	66 (5.1)	0.47
**Metabolic and nutritional diseases**	66 (10.3)	93(14.4)	159 (12.3)	0.025
Hypokalemia	39 (6.1)	68 (10.5)	107 (8.3)	0.004
Hypoproteinemia	35 (5.4)	40 (6.2)	75 (5.8)	0.58
**Nervous system disorders**	64 (10.0)	76 (11.7)	140 (10.8)	0.31
Headache	40 (6.2)	46 (7.1)	86 (6.7)	0.53
Dizziness	36 (5.6)	37 (5.7)	73 (5.7)	0.93
**Hepatobiliary diseases**	52 (8.1)	61 (9.4)	113 (8.8)	0.4
Liver function abnormalities	52 (8.1)	61 (9.4)	113 (8.8)	0.4
**Cardiovascular disorders**	51 (7.9)	42 (6.5)	93 (7.2)	0.31
Increased blood pressure	51 (7.9)	42 (6.5)	93 (7.2)	0.31
**Psychiatric disorders**	39 (6.1)	34 (5.3)	73 (5.7)	0.52
Insomnia	39 (6.1)	34 (5.3)	73 (5.7)	0.52
**Respiratory, chest and mediastinal diseases**	29 (4.5)	37 (5.7)	66 (5.1)	0.33
Cough	29 (4.5)	37 (5.7)	66 (5.1)	0.33

Data are presented as n (%)

Increased blood pressure was defined as a systolic blood pressure of 120–139 mmHg or a diastolic blood pressure of 80–89 mmHg

There were no clinically significant changes in vital signs and most clinical laboratory parameters between groups (Supplementary file). In a small number of patients there were changes in blood, urinary, and blood biochemistry parameters; however, these were associated with the underlying disease or were recorded as an AE. There were no clinically significant changes in electrocardiogram measurements for both groups. In total, there were 23 deaths in the study (cinepazide maleate group, *n* = 10; control group, *n* = 13), of which none were attributed to the study drug. Eighteen deaths were related to multiple organ dysfunctional syndrome, cerebral hernia, and acute myocardial infarction with the remaining five deaths of unknown cause.

## Discussion

This was a large-scale, multicenter, randomized, double-blind, placebo-controlled study that aimed to validate cinepazide maleate injection for the treatment of acute ischemic stroke in China. The results showed that, compared with placebo, a significantly higher proportion of patients treated with cinepazide maleate achieved an mRS score ≤ 2 and Barthel Index ≥95 at day 90, indicating that cinepazide maleate promotes post-stroke functional recovery and improves long-term activities of daily living (ADL) score compared with standard treatment.

During ischemia, adenosine is released in large quantities [21]. This is thought to ameliorate brain injury by reducing Ca^2+^ influx and lowering the presynaptic release of the excitotoxic neurotransmitter glutamate [21–23]. Adenosine and its receptors are attractive therapeutic targets for the treatment of stroke, although many selective A_1_ agonists cause sedation, bradycardia, and hypotension [24]. As a potentiator of adenosine A2 receptors, cinepazide maleate not only selectively potentiates the relaxing response of adenosine, it also prevents adenosine degradation and increases vasodilation via its effects on vascular endothelial function, and thus potentially reduces disability after stroke [10, 25, 26].

The efficacy of cinepazide maleate in the treatment of patients with acute carotid cerebral infarction has previously been reported in several randomized controlled trials [13–15]. For example, in a study by Liu et al., treatment with cinepazide maleate within 24 h significantly improved cerebral blood flow (*p* < 0.05) and the Barthel Index (*p* < 0.05) in patients with craniocerebral trauma compared with standard treatment [13]. In addition, Zhang et al. showed that, in patients treated with cinepazide maleate within 12 h of an acute carotid stroke, there was a significant improvement in the combined primary outcome (mRS level 0–2, Barthel Index ≥75, NIHSS score 0–1, and an NIHSS score that had dropped by > 8 points from baseline) on day 90 compared with standard treatment alone (*p* = 0.047) [14]. The mRS and Barthel Index assess global disability and ADL (including self-care and mobility), respectively, and both can be used to predict a patient’s independence [27]. In this study, the proportion of patients with an mRS ≤2 or a Barthel Index of ≥95 on day 90 were higher after cinepazide maleate treatment compared with standard treatment. These data are similar to those from previous clinical trials; therefore, it was concluded that cinepazide maleate injection improves neurological function, thus reducing disability and promoting functional recovery.

Overall, the safety profile of cinepazide maleate administration was similar to what has previously been reported in the literature [28–31]. Cinepazide maleate therapy has also previously been associated with AEs related to the blood system (e.g., leukopenia and neutropenia) [28]. The incidence of leukopenia was 0.4% in one study [29] and 0.2% in another [31]. In both studies there were no cases of agranulocytosis reported. In the present study, decreased white blood cells was only reported as an AE in four patients (0.6%) and three patients (0.5%) in the cinepazide maleate and control groups, respectively. Of these, only one case in the cinepazide maleate group was reported as a drug-related AE, and this was subsequently resolved without treatment. Leukopenia was only reported in one patient (0.2%) in the cinepazide maleate group, consistent with previous reports; however, this case was not considered related to cinepazide maleate therapy. Therefore, our data indicate that cinepazide maleate is well tolerated and has a safety profile similar to that reported previously.

The main limitation of this study is the lack of generalizability to other populations because the sample comprised only Chinese patients who had experienced a cerebral infarction caused by an acute internal carotid artery stroke. As such, patients who experienced an acute stroke that produced a disorder of consciousness were excluded. However, it is standard practice to exclude subjects with minor stroke from trials of stroke therapy owing to their high self-recovery capacity [32], and the threshold for minor stroke was set slightly higher in the present study than in overseas studies based on Chinese Food and Drug Administration guidelines. Thus, the findings are potentially very relevant for Chinese stroke patients outside of China and may support a need for regulatory applications and further trials in other countries. In addition, for a post-marketing confirmatory study, the follow-up period was relatively short; therefore, future studies are required to investigate the effect of cinepazide maleate injection on long-term recovery, although the results to 90 days are encouraging.

## Conclusions

The results of this study confirm that cinepazide maleate injection is safe and effective for the treatment of patients following acute ischemic stroke. Overall, cinepazide maleate injection was well tolerated with no novel safety issues reported.

### Supplementary information

**Additional file 1: Supplementary Table.** Laboratory results and vital signs for each group before and after treatment (safety set)

## Data Availability

The datasets used and/or analyzed during the current study are available from the corresponding author on reasonable request.

## Appendix

**Table 4:** The following ethics committees (including institutions that did not enroll patients) approved the study protocol: Reference numbers for each approval are provided

#	Ethics committee name	Reference number
1	Ethics Committee of Peking Union Medical College Hospital	KS2018077
2	Institutional Review Board of Chinese PLA General Hospital	C2016–023-05
3	Ethics Committee of Army General Hospital of PLA	BZEC2016-YW-001-03
4	Ethics Committee of Beijing Hospital	2016BJYYEC-105-05
5	Peking University Third Hospital Medical Science Research Ethics Committee	2017–007-04
6	Biomedical Research Ethics Committee of Peking University First Hospital	2016–07
7	Ethics Committee of Naval General Hospital	HZYW-YJ-2016-7-2
8	China Rehabilitation Research Center Medical Ethics Committee	2017–008
10	Tianjin Huanhu Hospital Medical Ethics Committee	2018–7
11	Ethical Committee of The Second Hospital of Hebei Medical University	2015R001846
12	Ethics Committee of Harrison International Peace Hospital	2016-03-08
13	Ethics Committee of Jinan Central Hospital	AF/SC-02/02.0
16	IRB of Shuguang Hospital affiliated with Shanghai University of TCM	2016–491-42
17	Ethics Committee of the Affiliated Hospital of Xuzhou Medical College	XYFY2016-YL016–15
18	Ethics Committee of the Second Affiliated Hospital of Nanjing Medical University	2016-YW-008-LP-01
19	Medical Ethics Committee of Zhejiang Hospital	2016–15G-X3
20	Ethics Committee of Affiliated Hospital of Hangzhou Normal University	2018–00000037
21	Ethics Committee of Sir Run Run Shaw Hospital of Zhejiang University School of Medicine	20,170,809–5
22	Ethics Committee of First Affiliated Hospital of Wenzhou Medical College	2018–058
23	Ethical Committee of The Jiangxi Provincial. People’s Hospital	2018–11
24	Ethics Committee of the Third Affiliated Hospital of Nanchang University	2016–001
26	YiChang Central People’s Hospital Medical Ethics Committee	2016–005-11
27	Ethics Committee of Huangshi Central Hospital	2016-SJN-004
28	Ethics Committee of Xiangya Hospital Central South University	201,708,085
29	Ethics Committee of the Third Hospital of Changsha	2016EC-006
30	Ethics Committee of First People’s Hospital of Yueyang	2016–006–2018-02
31	Ethic committee of the First Affiliated Hospital of Xiamen University	XMYY-2016Y016–05
32	Medical Ethics Committee of Zhongshan Hospital Affiliated to Xiamen University	XMZSIRB2018–017
33	Medical Ethics Committee of Sun Yat-Sen Memorial Hospital, Sun Yat-Sen University	2019–03
34	Medical ethics committee branch of Dongguan People’s Hospital	DRYA2016–008-B1
35	Ethics Committee of Liuzhou Worker’s Hospital	201,615-PL1
36	Ethical Committee of Hainan provincial people’s Hospital	2017–100
38	Ethics Committee of the General Hospital of Shenyang Military Region of the Chinese People’s Liberation Army	2016-51-2
39	Ethics Committee of the People’s Hospital of Jilin Province	2016-Y-016
40	Ethics Committee of the Affiliated Hospital of Yanbian University	2016–008-09
41	Ethics Committee of Inner Mongolia Baogang Hospital	2016-BL-12
42	Ethics Committee of Inner Mongolia Autonomous Region People’s Hospital	YWLCSYLL2016–014-06
43	Ethics Review Committee of Shaanxi Provincial People’s Hospital	2016Y004
44	Ethics Committee of Ningxia Medical University General Hospital	2018–17
47	Ethics Committee of Xuan Wu Hospital of Capital Medical University	2016–022-2
48	The Second Hospital of Shanxi Medical University Ethics Committee	2017-KS-087
49	Tianjin First Central Hospital Clinical Research Ethics Committee	2019–010
50	Ethics Committee of the First Hospital of Jilin University	2018–161,123–224-1
51	Ethics Committee of the Hunan Provincial People’s Hospital	2016–07.1
52	Ethics Committee of the Second Affiliated Hospital of University of South China	201,806–01-034
53	Ethics Committee of Xuzhou Central Hospital	XZXY-LY-20180502-2,016,010
55	Ethics Committee of Pingxiang People’s Hospital	2016D010-F08
56	Ethics Committee of Affiliated Hospital of Nantong University	2016-Y010-X02
58	Ethics Committee of The First Affiliated Hospital of Soochow University	2017–061
59	Ethics Committee of Baotou Central Hospital	2018-13H
60	Medical Ethics Committee of Lishui Municipal Central Hospital	2017-02-03
61	Ethics Committee of the Affiliated Hospital of Chengde Medical College	2018LL-K001
62	Ethics Committee of the second Hospital of Jilin University	2017–006
63	Ethics Committee of Jilin Neuropsychiatric Hospital	2018--03
65	Ethics Committee of Meihekou City Central Hospital	2017-SNK-001
66	Medical Ethics Committee of the Fourth Affiliated Hospital of Harbin Medical University	2017–006
67	Quzhou People’s Hospital Ethics Committee	2017-1-2
68	Ethics Committee of Chenzhou First People’s Hospital	20,170,629
70	Medical Ethics Committee of Peking University Shougang Hospital	IRB-2017-023-11
71	Ethics Committee of Taizhou People’s Hospital	YW201700620
72	Ethics Committee of Huai’an Second People’s Hospital	HEYL-P-2017-01-03
73	Ethic Committee of The Third Affiliated Hospital of Southern Medical University	2017-08-2
74	Handan Center Hospital Research Ethics Committee	2017–001-02
75	Ethics Committee of General Hospital of Xuzhou Mining Group	2018–042301
76	Ethics Committee of Zhengzhou First People’s Hospital	2017–01-001
77	Ethics Committee of Cangzhou Hospital of integrated Traditional Chinese and Western Medicine of Hebei Province	2,018,006
78	Ethics Committee of the First Affiliated Hospital Of Henan University	No reference number available
79	Ethics Committee of Luohe Central Hospital	IEC-C-008-A07-V1.1
80	Medical Ethics Committee of Dalian Municipal Central Hospital	2017–013-02
81	Ethics Committee of Tonghua Municipal Central Hospital	2018–03

## Abbreviations

AE: Adverse event
CI: Confidence interval
mRS: modified Rankin scale
NIHSS: National Institutes of Health Stroke Scale
OR: Odds ratio
SD: Standard deviation
