# Supplementary material for: Efficacy and safety of cinepazide maleate injection in patients with acute ischemic stroke: a multicenter, randomized, double-blind, placebo-controlled trial
Source: BMC Neurol. 2020 Jul 14;20:282. doi: 10.1186/s12883-020-01844-8 (PMC7359492; doi:10.1186/s12883-020-01844-8)
Supplement: Supplementary file 1 — Additional file 1: Supplementary Table. Laboratory results and vital signs for each group before and after treatment (safety set) [file 12883_2020_1844_MOESM1_ESM.doc]

**Supplementary Table. Laboratory results and vital signs for each group before and after treatment (safety set)**

| **Parameter** | **Experimental group (N=643)** | **Control group (N=648)** | **Total (N=1291)** |
| --- | --- | --- | --- |
| **Red blood cell count (×10^12/L)** |  |  |  |
| Baseline |  |  |  |
| Number of cases (missing) | 643 (0) | 647 (1) | 1290 (1) |
| Mean (standard deviation) | 4.723 (0.5418) | 4.688 (0.5678) | 4.705 (0.5551) |
| Median (Q1, Q3) | 4.70 (4.37, 5.07) | 4.69 (4.31, 5.01) | 4.70 (4.36, 5.04) |
| Minimum, maximum | 2.87, 6.81 | 2.61, 6.67 | 2.61, 6.81 |
| 90th day |  |  |  |
| Number of cases (missing) | 548 (95) | 550 (98) | 1098 (193) |
| Mean (standard deviation) | 4.603 (0.5333) | 4.587 (0.4880) | 4.595 (0.5110) |
| Median (Q1, Q3) | 4.60 (4.28, 4.95) | 4.59 (4.29, 4.89) | 4.60 (4.28, 4.91) |
| Minimum, maximum | 2.44, 7.18 | 2.27, 5.89 | 2.27, 7.18 |
| 90th day − baseline |  |  |  |
| Number of cases (missing) | 548 (95) | 549 (99) | 1097 (194) |
| Mean (standard deviation) | −0.132 (0.4818) | −0.117 (0.4408) | −0.124 (0.4616) |
| Median (Q1, Q3) | −0.12 (−0.40, 0.15) | −0.09 (−0.39, 0.18) | −0.10 (−0.39, 0.16) |
| Minimum, maximum | −1.92, 1.97 | −2.00, 1.26 | −2.00, 1.97 |
| Paired t-test (P value) | −6.43 (<.0001) | −6.20 (<.0001) | −8.93 (<.0001) |
|  |  |  |  |
| **White blood cell count (×10^9/L)** |  |  |  |
| Baseline |  |  |  |
| Number of cases (missing) | 643 (0) | 647 (1) | 1290 (1) |
| Mean (standard deviation) | 7.913 (2.6521) | 7.765 (2.5183) | 7.839 (2.5859) |
| Median (Q1, Q3) | 7.54 (6.18, 9.00) | 7.40 (5.88, 9.16) | 7.50 (6.08, 9.08) |
| Minimum, maximum | 2.94, 29.77 | 3.48, 22.23 | 2.94, 29.77 |
| 90th day |  |  |  |
| Number of cases (missing) | 548 (95) | 550 (98) | 1098 (193) |
| Mean (standard deviation) | 6.998 (1.8197) | 6.820 (1.7692) | 6.909 (1.7960) |
| Median (Q1, Q3) | 6.71 (5.74, 7.93) | 6.68 (5.62, 7.80) | 6.70 (5.69, 7.81) |
| Minimum, maximum | 3.10, 14.20 | 2.85, 15.68 | 2.85, 15.68 |
| 90th day − baseline |  |  |  |
| Number of cases (missing) | 548 (95) | 549 (99) | 1097 (194) |
| Mean (standard deviation) | −0.887 (2.5125) | −0.859 (2.2403) | −0.873 (2.3791) |
| Median (Q1, Q3) | −0.61 (−1.93, 0.70) | −0.60 (−1.90, 0.50) | −0.60 (−1.91, 0.62) |
| Minimum, maximum | −16.30, 6.80 | −13.89, 7.22 | −16.30, 7.22 |
| Paired t-test (P value) | −8.26 (<.0001) | −8.98 (<.0001) | −12.15 (<.0001) |
|  |  |  |  |
| **Hemoglobin (g/L)** |  |  |  |
| Baseline |  |  |  |
| Number of cases (missing) | 643 (0) | 647 (1) | 1290 (1) |
| Mean (standard deviation) | 143.642 (16.3060) | 141.683 (16.7391) | 142.660 (16.5473) |
| Median (Q1, Q3) | 144.00 (134.00, 155.00) | 142.00 (132.00, 152.00) | 143.00 (133.00, 153.00) |
| Minimum, maximum | 92.00, 197.00 | 72.00, 188.00 | 72.00, 197.00 |
| 90th day |  |  |  |
| Number of cases (missing) | 548 (95) | 550 (98) | 1098 (193) |
| Mean (standard deviation) | 138.777 (15.6800) | 137.822 (14.6063) | 138.299 (15.1523) |
| Median (Q1, Q3) | 139.00 (129.00, 149.00) | 138.00 (129.00, 147.00) | 138.00 (129.00, 148.00) |
| Minimum, maximum | 47.00, 191.00 | 75.00, 181.00 | 47.00, 191.00 |
| 90th day − baseline |  |  |  |
| Number of cases (missing) | 548 (95) | 549 (99) | 1097 (194) |
| Mean (standard deviation) | −5.223 (14.2861) | −4.455 (13.1425) | −4.839 (13.7248) |
| Median (Q1, Q3) | −4.00 (−14.00, 4.00) | −4.00 (−13.00, 4.00) | −4.00 (−13.00, 4.00) |
| Minimum, maximum | −68.00, 50.00 | −67.00, 42.00 | −68.00, 50.00 |
| Paired t-test (P value) | −8.56 (<.0001) | −7.94 (<.0001) | −11.68 (<.0001) |
|  |  |  |  |
| **Platelet count (×10^9/L)** |  |  |  |
| Baseline |  |  |  |
| Number of cases (missing) | 643 (0) | 647 (1) | 1290 (1) |
| Mean (standard deviation) | 224.136 (59.5546) | 222.181 (59.0607) | 223.155 (59.2924) |
| Median (Q1, Q3) | 215.00 (186.00, 258.00) | 220.00 (179.00, 257.00) | 218.00 (182.00, 258.00) |
| Minimum, maximum | 88.00, 566.00 | 69.00, 605.00 | 69.00, 605.00 |
| 90th day |  |  |  |
| Number of cases (missing) | 548 (95) | 550 (98) | 1098 (193) |
| Mean (standard deviation) | 232.482 (63.1889) | 232.740 (63.1415) | 232.611 (63.1365) |
| Median (Q1, Q3) | 229.00 (188.50, 269.50) | 231.00 (185.00, 275.00) | 230.00 (187.00, 273.00) |
| Minimum, maximum | 73.00, 548.00 | 92.00, 627.00 | 73.00, 627.00 |
| 90th day − baseline |  |  |  |
| Number of cases (missing) | 548 (95) | 549 (99) | 1097 (194) |
| Mean (standard deviation) | 7.589 (46.1664) | 10.339 (49.3622) | 8.965 (47.7905) |
| Median (Q1, Q3) | 6.00 (−17.00, 32.00) | 10.00 (−17.00, 36.00) | 8.00 (−17.00, 35.00) |
| Minimum, maximum | −160.00, 201.00 | −346.00, 187.00 | −346.00, 201.00 |
| Paired t-test (P value) | 3.85 (0.0001) | 4.91 (<.0001) | 6.21 (<.0001) |
|  |  |  |  |
| **Percent lymphocytes (%)** |  |  |  |
| Baseline |  |  |  |
| Number of cases (missing) | 643 (0) | 647 (1) | 1290 (1) |
| Mean (standard deviation) | 22.666 (9.5923) | 22.598 (8.9762) | 22.632 (9.2849) |
| Median (Q1, Q3) | 22.10 (17.00, 28.50) | 22.20 (16.00, 28.70) | 22.20 (16.60, 28.50) |
| Minimum, maximum | 0.11, 99.24 | 0.09, 56.40 | 0.09, 99.24 |
| 90th day |  |  |  |
| Number of cases (missing) | 548 (95) | 550 (98) | 1098 (193) |
| Mean (standard deviation) | 27.734 (7.6380) | 28.040 (7.8991) | 27.888 (7.7678) |
| Median (Q1, Q3) | 27.80 (22.10, 32.75) | 27.40 (22.50, 33.40) | 27.65 (22.20, 32.90) |
| Minimum, maximum | 0.20, 48.10 | 0.14, 57.80 | 0.14, 57.80 |
| 90th day − baseline |  |  |  |
| Number of cases (missing) | 548 (95) | 549 (99) | 1097 (194) |
| Mean (standard deviation) | 4.864 (9.5223) | 5.245 (9.2156) | 5.055 (9.3677) |
| Median (Q1, Q3) | 4.15 (−0.60, 10.05) | 4.60 (−1.00, 10.20) | 4.40 (−0.80, 10.10) |
| Minimum, maximum | −68.94, 39.54 | −27.70, 42.63 | −68.94, 42.63 |
| Paired t-test (P value) | 11.96 (<.0001) | 13.34 (<.0001) | 17.87 (<.0001) |
|  |  |  |  |
| **Percent neutrophils (%)** |  |  |  |
| Baseline |  |  |  |
| Number of cases (missing) | 643 (0) | 647 (1) | 1290 (1) |
| Mean (standard deviation) | 68.068 (13.5682) | 68.525 (12.7156) | 68.297 (13.1444) |
| Median (Q1, Q3) | 69.30 (61.80, 76.00) | 69.40 (62.00, 77.00) | 69.35 (61.80, 76.20) |
| Minimum, maximum | 0.44, 93.50 | 0.56, 93.20 | 0.44, 93.50 |
| 90th day |  |  |  |
| Number of cases (missing) | 548 (95) | 550 (98) | 1098 (193) |
| Mean (standard deviation) | 62.698 (8.9855) | 62.644 (8.7028) | 62.671 (8.8410) |
| Median (Q1, Q3) | 62.70 (57.41, 68.75) | 63.15 (57.50, 68.40) | 62.80 (57.50, 68.50) |
| Minimum, maximum | 0.68, 87.20 | 0.78, 87.81 | 0.68, 87.81 |
| 90th day − baseline |  |  |  |
| Number of cases (missing) | 548 (95) | 549 (99) | 1097 (194) |
| Mean (standard deviation) | −4.886 (14.0967) | −5.657 (12.6022) | −5.272 (13.3691) |
| Median (Q1, Q3) | −5.00 (−12.35, 1.00) | −5.10 (−12.70, 1.20) | −5.00 (−12.50, 1.10) |
| Minimum, maximum | −86.32, 79.19 | −47.90, 69.25 | −86.32, 79.19 |
| Paired t-test (P value) | −8.11 (<.0001) | −10.52 (<.0001) | −13.06 (<.0001) |
|  |  |  |  |
| **Absolute neutrophil count (×10^9/L)** |  |  |  |
| Baseline |  |  |  |
| Number of cases (missing) | 643 (0) | 647 (1) | 1290 (1) |
| Mean (standard deviation) | 5.608 (2.5709) | 5.513 (2.3666) | 5.560 (2.4701) |
| Median (Q1, Q3) | 5.04 (3.92, 6.60) | 5.10 (3.80, 6.65) | 5.08 (3.89, 6.61) |
| Minimum, maximum | 0.02, 26.59 | 1.76, 19.41 | 0.02, 26.59 |
| 90th day |  |  |  |
| Number of cases (missing) | 548 (95) | 550 (98) | 1098 (193) |
| Mean (standard deviation) | 4.437 (1.4833) | 4.329 (1.4389) | 4.383 (1.4615) |
| Median (Q1, Q3) | 4.20 (3.41, 5.21) | 4.14 (3.33, 5.09) | 4.17 (3.40, 5.10) |
| Minimum, maximum | 1.60, 12.32 | 1.36, 12.05 | 1.36, 12.32 |
| 90th day − baseline |  |  |  |
| Number of cases (missing) | 548 (95) | 549 (99) | 1097 (194) |
| Mean (standard deviation) | −1.113 (2.5049) | −1.103 (2.1836) | −1.108 (2.3486) |
| Median (Q1, Q3) | −0.69 (−2.10, 0.30) | −0.73 (−2.11, 0.25) | −0.71 (−2.10, 0.26) |
| Minimum, maximum | −18.98, 6.20 | −14.75, 5.37 | −18.98, 6.20 |
| Paired t-test (P value) | −10.40 (<.0001) | −11.83 (<.0001) | −15.62 (<.0001) |
|  |  |  |  |
| **Body temperature (armpit) (ºC)** |  |  |  |
| Baseline |  |  |  |
| Number of cases (missing) | 643 (0) | 647 (1) | 1290 (1) |
| Mean (standard deviation) | 36.52 (0.337) | 36.53 (0.331) | 36.52 (0.334) |
| Median (Q1, Q3) | 36.5 (36.3, 36.7) | 36.5 (36.3, 36.7) | 36.5 (36.3, 36.7) |
| Minimum, maximum | 35.5, 38.4 | 35.3, 38.5 | 35.3, 38.5 |
| 90th day |  |  |  |
| Number of cases (missing) | 568 (75) | 565 (83) | 1133 (158) |
| Mean (standard deviation) | 36.52 (0.334) | 36.51 (0.306) | 36.51 (0.320) |
| Median (Q1, Q3) | 36.5 (36.3, 36.7) | 36.5 (36.3, 36.6) | 36.5 (36.3, 36.7) |
| Minimum, maximum | 35.5, 38.4 | 35.3, 37.9 | 35.3, 38.4 |
| 90th day − baseline |  |  |  |
| Number of cases (missing) | 566 (77) | 561 (87) | 1127 (164) |
| Mean (standard deviation) | −0.11 (0.388) | −0.12 (0.359) | −0.12 (0.374) |
| Median (Q1, Q3) | −0.1 (−0.3, 0.1) | −0.1 (−0.3, 0.1) | −0.1 (−0.3, 0.1) |
| Minimum, maximum | −2.3, 1.5 | −1.6, 1.0 | −2.3, 1.5 |
| Paired t-test (P value) | −6.6 (<.0001) | −8.2 (<.0001) | −10.4 (<.0001) |
|  |  |  |  |
| **Pulse (beats/min)** |  |  |  |
| Baseline |  |  |  |
| Number of cases (missing) | 643 (0) | 647 (1) | 1290 (1) |
| Mean (standard deviation) | 75.1 (10.44) | 75.1 (10.70) | 75.1 (10.56) |
| Median (Q1, Q3) | 75 (69, 80) | 75 (68, 80) | 75 (68, 80) |
| Minimum, maximum | 50, 136 | 44, 110 | 44, 136 |
| 90th day |  |  |  |
| Number of cases (missing) | 568 (75) | 565 (83) | 1133 (158) |
| Mean (standard deviation) | 75.1 (10.41) | 74.9 (10.51) | 75.0 (10.46) |
| Median (Q1, Q3) | 76 (69, 80) | 75 (68, 80) | 75 (69, 80) |
| Minimum, maximum | 50, 136 | 44, 108 | 44, 136 |
| 90th day − baseline |  |  |  |
| Number of cases (missing) | 567 (76) | 562 (86) | 1129 (162) |
| Mean (standard deviation) | 0.0 (11.83) | 0.7 (12.09) | 0.4 (11.96) |
| Median (Q1, Q3) | 0 (−7, 7) | 1 (−7, 8) | 0 (−7 ,8) |
| Minimum, maximum | −59, 61 | −29, 55 | −59, 61 |
| Paired t-test (P value) | 0 (0.9491) | 1 (0.1594) | 1 (0.2940) |
|  |  |  |  |
| **Respiration (breaths/min)** |  |  |  |
| Baseline |  |  |  |
| Number of cases (missing) | 642 (1) | 647 (1) | 1289 (2) |
| Mean (standard deviation) | 18.7 (1.44) | 18.7 (1.47) | 18.7 (1.46) |
| Median (Q1, Q3) | 18 (18, 20) | 18 (18, 20) | 18 (18, 20) |
| Minimum, maximum | 12, 22 | 12, 26 | 12, 26 |
| 90th day |  |  |  |
| Number of cases (missing) | 567 (76) | 565 (83) | 1132 (159) |
| Mean (standard deviation) | 18.7 (1.44) | 18.7 (1.48) | 18.7 (1.46) |
| Median (Q1, Q3) | 18 (18, 20) | 18 (18, 20) | 18 (18, 20) |
| Minimum, maximum | 12, 22 | 12, 26 | 12, 26 |
| 90th day − baseline |  |  |  |
| Number of cases (missing) | 566 (77) | 561 (87) | 1127 (164) |
| Mean (standard deviation) | −0.2 (1.69) | −0.3 (1.85) | −0.2 (1.77) |
| Median (Q1, Q3) | 0 (−1, 1) | 0 (−2, 1) | 0 (−1, 1) |
| Minimum, maximum | −7, 5 | −8, 10 | −8, 10 |
| Paired t-test (P value) | −3 (0.0034) | −4 (0.0005) | −5 (<.0001) |
|  |  |  |  |
| **Systolic blood pressure (mmHg)** |  |  |  |
| Baseline |  |  |  |
| Number of cases (missing) | 643 (0) | 648 (0) | 1291 (0) |
| Mean (standard deviation) | 148.2 (20.66) | 147.5 (19.66) | 147.9 (20.16) |
| Median (Q1, Q3) | 147 (132, 163) | 147 (134, 160) | 147 (133, 161) |
| Minimum, maximum | 98, 198 | 81, 199 | 81, 199 |
| 90th day |  |  |  |
| Number of cases (missing) | 568 (75) | 566 (82) | 1134 (157) |
| Mean (standard deviation) | 148.0 (20.32) | 147.4 (19.54) | 147.7 (19.92) |
| Median (Q1, Q3) | 146 (132, 163) | 147 (134, 160) | 146 (133, 161) |
| Minimum, maximum | 98, 198 | 81, 199 | 81, 199 |
| 90th day − baseline |  |  |  |
| Number of cases (missing) | 567 (76) | 564 (84) | 1131 (160) |
| Mean (standard deviation) | −13.7 (21.89) | −11.6 (21.64) | −12.6 (21.78) |
| Median (Q1, Q3) | −12 (−28, 0) | −10 (−25, 3) | −10 (−26, 2) |
| Minimum, maximum | −86, 47 | −77, 50 | −86, 50 |
| Paired t-test (P value) | −15 (<.0001) | −13 (<.0001) | −19 (<.0001) |
|  |  |  |  |
| **Diastolic blood pressure (mmHg)** |  |  |  |
| Baseline |  |  |  |
| Number of cases (missing) | 643 (0) | 648 (0) | 1291 (0) |
| Mean (standard deviation) | 85.7 (11.62) | 86.0 (11.57) | 85.9 (11.59) |
| Median (Q1, Q3) | 85 (79, 94) | 86 (78, 94) | 85 (78, 94) |
| Minimum, maximum | 52, 124 | 48, 120 | 48, 124 |
| 90th day |  |  |  |
| Number of cases (missing) | 568 (75) | 566 (82) | 1134 (157) |
| Mean (standard deviation) | 85.6 (11.47) | 85.9 (11.45) | 85.8 (11.46) |
| Median (Q1, Q3) | 85 (79, 94) | 86 (78, 94) | 85 (78, 94) |
| Minimum, maximum | 52, 122 | 48, 120 | 48, 122 |
| 90th day − baseline |  |  |  |
| Number of cases (missing) | 567 (76) | 564 (84) | 1131 (160) |
| Mean (standard deviation) | −4.3 (12.42) | −3.7 (13.35) | −4.0 (12.89) |
| Median (Q1, Q3) | −4 (−12, 4) | −4 (−12, 5) | −4 (−12, 4) |
| Minimum, maximum | −53, 42 | −42, 37 | −53, 42 |
| Paired t-test (P value) | −8 (<.0001) | −6 (<.0001) | −10 (<.0001) |
|  |  |  |  |
